# Monitoring training status using novel submaximal test

**DOI:** 10.1186/2052-1847-7-S1-O4

**Published:** 2015-08-11

**Authors:** M J Hofmijster, G J J van de Plasse, A M C van Beijsterveldt, J H Stubbe

**Affiliations:** 1 Faculty of Human Movement Sciences, VU Amsterdam, the Netherlands; 2 Hogeschool van Amsterdam, Amsterdam, the Netherlands; 3 TNO Life Style, Leiden, the Netherlands

## Introduction

Rowers in their first year of competition have a high risk of overtraining, caused by a disbalance in training load and recovery. A typical week in the rowing season consists of 5-8 training sessions, and many first year (“freshmen”) rowers have little previous experience in competitive sports. The risk is further increased by the fact that personalization of training load is difficult and that coaches are often inexperienced. In this study we investigated whether a standardized submaximal exercise rowing test (SERT) can be used as a tool to monitor training status and as such can help to prevent overtraining.

## Methods

For a period of four weeks, the weekly training load of members of one lightweight men’s freshmen crew (eight rowers) was quantified by summating heart rate scores (Edwards, 1993). Rowers undertook the SERT on a rowing ergometer (Concept 2, USA) on a weekly basis. The SERT adapted from the LSCT (Lamberts, 2011) and yields values for power output at 70, 80 and 90% of maximum heart rate, heart rate recovery (HRR, defined as the drop in heart rate in one minute after cessation of exercise) and rate of perceived exertion (RPE) on a 0-10 point scale (Foster 2001).

## Results

One participant dropped out because he showed signs of overtraining already at the start of the experiment. For the remaining seven rowers, training load was significantly higher in weeks 2 and 3 compared to weeks 1 and 4 (P<0.01). Consequently, these weeks were categorized as ‘heavy weeks’ (H) whereas weeks 1 and 4 were categorized as ‘light to moderate’ weeks (L). HRR was shown to be significantly lower during the heavy weeks (L: 53.8±8.48, H: 50.6±6.50, P<0.05), suggesting fatigue or inaccurate recovery. No differences were found in power values or RPE scores.

## Discussion

Obtaining relevant information on training status of individual team members can prove to be difficult in crew rowing. This study indicates that a standardized training such as the SERT helps to monitor individual rowers, especially when HRR is obtained. A sudden drop in HRR is as an early warning sign and may lead a coach to adapt the training schedule. Furthermore, the participants rated the SERT with 3.8 on the 0-10 RPE scale, indicating that the SERT is suitable to be performed on a frequent basis without interfering with the daily training. Future research should point out whether occurrence of overtraining would indeed be lower with frequent monitoring of training status.
